# Medical History from the Earliest Times

**Published:** 1893-05-20

**Authors:** E. T. Withington


### Medical History from the Earliest Times.

By E. T. Withington, M.A., M.B. Oxon.

### LV.—THE CHEMICAL SCHOOL OF MEDICINE.

The origin of the so-called iatro-chemical school is often traced to Van Helmont, but its doctrines may be rather contrasted than compared with those of the Flemish physician, which are far more closely allied to the later vitalistic theories. The seventeenth century, like our own, was an age of physical science, a character due partly to a reaction from the mysticism of the preceding period, but especially to the influence of the discoveries of Harvey and Galileo, and the teaching of Bacon and Descartes. Bacon, by formulating the principles of inductive science, had pointed out the true path of progress between the barren desert of Aristotelean dialectics on the one side, and the mist-enveloped swamp of Neo-Platonic theosophy on the other; but his influence on medicine is probably less important than that of Descartes. It seemed to the French philosopher that the universe contains two distinct things—matter, with the characteristic property of extension; and mind, with the characteristic property of thought, between which he held that there can be no conceivable connection; and he declared that the only proof of the existence of mind is conscious thought—"Cogito ergo sum." But all the processes of life may go on without conscious thought, therefore vital activity is of a purely material character; and Descartes boldly asserted that animals are merely self-acting machines or automata, while man is a similar machine with a mind behind it and sometimes acting upon it. This Cartesian dualism, as it may be called, dominates the medical theories of the seventeenth and part of the eighteenth centuries, as well as those of our own day, but it is the very reverse of the doctrine of Paracelsus and Van Helmont, who held that matter and mind are intimately connected, and that all the processes of nature are lower or higher, less or more self-conscious manifestations of one universal life. The physicians of the seventeenth century made two attempts to solve the problems of life and disease on purely materialistic principles, one by the aid of chemistry, the other by the aid of mechanics, and thus arose two schools or systems of medicine known respectively as the iatro-chemical and the iatro-mechanical.

The foremost and most typical representative of the former school is Francis de le Boe (Sylvius) (1614-72), whose chemistry, however, is based not upon that of the Paracelsists with its arcana and quintessences, but upon the more sober observations and experiments of their great opponents, Libavius and Glauber. And he contrasts with his predecessors in his purpose as well as in his method. Paracelsus and Van Helmont had attempted to overthrow Galenism, and to replace it by new systems evolved largely from their own inner consciousness; Sylvius, on the contrary, strove to re-establish the humoral pathology on a firm basis of chemical facts, and he may not inappropriately be called "the last of the Galenists."

Sylvius commences with principles worthy of a disciple of Harvey, Bacon, and Descartes. Nothing, he says, is to be admitted as true in medicine or natural science unless experience has confirmed and shown it to be true. As regards medicine, this experience may be classed under three heads: (1) anatomical, (2) chemical, (3) clinical; but he warns his pupils against falling into the error of the old Empirics, that of drawing conclusions too hastily, and proposes to divide his results into conjectures, suspicions, opinions, and conclusions, according to their relative scientific value. Medicine, he declares, still rests mainly upon evidence of the first kind, and, though a progressive art, does not yet deserve the name of science. Its firmest pillars are anatomy, chemistry, and Harvey's doctrine of the circulation, which he vigorously defends against all remaining adversaries.

From such a preface great results might have been expected, but unfortunately even men of scientific mind are apt to give the rank of opinions, if not of conclusions, to theories of their own, which, if put forward by others, they would justly class among conjectures; and contempt for authority, though frequently leading to new truth, may also give rise to error. So now it was with the Leyden Professor. Among the matters in which most follow authority rather than reason is, says Sylvius, the doctrine that the bile is secreted by the liver, and passes thence into the gall-bladder. Careful anatomical observation has convinced him that this is absurd, and that the bile is secreted directly from the cystic artery into the gall-bladder, whence it passes partly into the intestine, and partly through the liver and vena cava into the heart. Here it meets with the acid lymph, brought by the thoracic duct and superior vena cava, and the combination of the acid and alkaline fluids produces a mild fermentation, the source at once of the bodily heat and the diastole of the heart. If either the acidity of the lymph, or the alkalinity of the bile be increased, the fermentation becomes more active, and we get increased heat and exaggerated action of the heart, or, in other words, "fever." Excessive increase or decrease of this cardiac fermentation causes death, and he explains the rapid collapse in "cholera" by the fact that all the bile is expelled by the intestine, and none left to go to the heart. The active parts of the body are the fluids or humours, the solids serving chiefly to contain them ("partes continentes"), and most diseases are due to an "acridity" of some humour, which may have become either too acid or too alkaline. Sylvius modestly admits that the above theories are only "opinions," but he nevertheless makes them the basis of his whole medical system.

In his therapeutic doctrines we find equally admirable principles, followed by a far less satisfactory application. The indications for treatment are, according to Sylvius, four in number—vital, curative, preservative, and symptomatic, or, in other words, to sustain the vitality, to cure the disease, to remove the predisposing cause, and to counteract any urgent symptoms. But in practice he reduces them to two—to suppress, by the use of narcotics, any violent symptoms which tend to exhaust the patient, and to counteract the excessive acidity or alkalinity by the use of contraries. The most common cause of disease, he considers, is "acidity," therefore, when in doubt, give an alkali.

A system which thus combined the prestige of the humoral pathology with the simplicity of the ancient methodism, and the interest of the new chemistry, was sure to be widely accepted, and traces not only of the theories but even of the prescriptions of Sylvius survived almost to our own times. The most famous of the latter was the "Elixir Proprietatis," which was largely used up to the middle of this century, and which closely resembled the "aromatic sulphuric acid," or elixir of vitriol of our present pharmacopœia.

But Sylvius has other and better claims to remembrance than that of being founder of a superficial and one-sided medical system. His name is justly immortalised in the human brain, the structure of which he did much to elucidate, and, above all, it is from him that we date the permanent establishment of clinical teaching in public hospitals. Bedside instruction had been given, as we have seen, in classical times, in the ancient Nestorian and Arabic schools, and by medical practitioners such as Lanfranc; and the system had been revived for a short period during the renaissance at Padua, when students from every part of Europe flocked to that famous university. But it is to the little infirmary of Leyden, with its twelve beds, that we must look for the true origin of the modern practice of "walking the hospitals." Writing in 1664, Sylvius observes, "I have led my pupils by the hand to medical practice, using a method unknown at Leyden, or perhaps elsewhere, *i.e.*, taking them daily to visit the sick at the public hospital. There I have put the symptoms of disease before their eyes; have let them hear the complaints of the patients, and have asked them their opinions as to the causes and rational treatment of each case, and the reasons for those opinions. Then I have given my own judgment on every point. Together with me they have seen the happy results of treatment when God has granted to our cares a restoration of health; or they have assisted in examining the body when the patient has paid the inevitable tribute to death."

The English representative of the chemical school of medicine was Thomas Willis (1622-75), whose name, like that of Sylvius, is best known from his researches on the structure and blood supply of the brain. But he is superior in many ways to his Flemish contemporary. His observations on the phenomena of disease and the action of drugs are second only (in that age) to those of Sydenham; his speculations on the part played in pathology by the nervous system or "animal spirits" anticipate some of the best results of the Vitalistic school, and his chemical theories, though necessarily imperfect, are less shallow and one-sided than those of Sylvius. According to Willis, the processes which take place in the animal organism are forms of "fermentation," which he defines as "an internal motion of the particles of any body, tending either to the perfection of the same body or because of its change into another," in short, what we now call "metabolism." Health and disease depend upon the normal or abnormal course of these fermentations, and the physician may be compared to a brewer or vintner, whose business is to watch the process, and to prevent or correct any irregularities. But Willis and his writings hardly received the attention they deserved, for the countrymen of Harvey and Newton were less attracted by chemical theories, than by the rival doctrines of the so-called mathematical or mechanical school of which we must now give a brief outline.

